# Tissue-Specific Profiling of Biflavonoids in Ginkgo (*Ginkgo biloba* L.)

**DOI:** 10.3390/plants12010147

**Published:** 2022-12-28

**Authors:** Marija Kovač Tomas, Iva Jurčević, Dunja Šamec

**Affiliations:** Department of Food Technology, University North, Trg Dr. Žarka Dolinara 1, 48000 Koprivnica, Croatia

**Keywords:** amentoflavone, bilobetin, ginkgetin, isoginkgetin, sciadopitysin, ginkgo, tissue-specific profiling, HPLC-DAD

## Abstract

Biflavonoids are flavonoid dimers that are much less studied than monomeric flavonoids. Their precise distribution among plants and their role in plants is still unknown. Here, we have developed a HPLC-DAD method that allows us to separate and simultaneously determine the five major biflavonoids (amentoflavone, bilobetin, ginkgetin, isoginkgetin, and sciadopitysin) in ginkgo (*Ginkgo biloba* L.). We performed tissue-specific profiling of biflavonoids in ten different plant parts: tree bark, twigs bark, twigs without bark, buds, leaf petioles, leaf blades, seed stalks, sarcotesta, nutshells, and kernels. We did not detect biflavonoids in plant parts not in direct contact with the environment (twigs without bark, nutshells, and kernels). We found the highest total biflavonoids content in leaves, where sciadopitysin was predominant. In contrast, in the bark, amentoflavone was the predominant biflavonoid, suggesting that more methylated biflavonoids accumulate in leaves and seeds. This is probably related to their biological function, which remains to be determined.

## 1. Introduction

Flavonoids are a large, and to date, most studied group of plant metabolites [1]. Flavonoids have a 15 carbon- atom flavone skeleton, C6-C3-C6, with two benzene rings (A and B) joined by a trinuclear pyran ring (C). The position of the B catechol ring on the C pyran ring, as well as the number and position of hydroxyl groups on the catechol group of the B ring, have a major influence on the chemical properties and biological activity of flavonoids [1,2,3]. In addition, flavonoids can be conjugated, glycosylated, or methylated, which also affects their biological properties and function in plants [1]. Flavonoid dimers, known as biflavonoids, consist of two monomeric flavonoids via a direct link or a linear linker. According to He et al. [4], nearly 600 different biflavonoids are known in angiosperms, ferns, gymnosperms, and bryophytes. Most of them are found in plants used in traditional medicine and are considered important factors in the health benefits of these plants [3,5,6]. However, the role of biflavonoids in plants is poorly studied. Based on their biological activity and localization in leaves [7,8], there are indications that they may be involved in protecting plants from pests and predators and in photosynthesis regulation [9].

**Figure 1:**
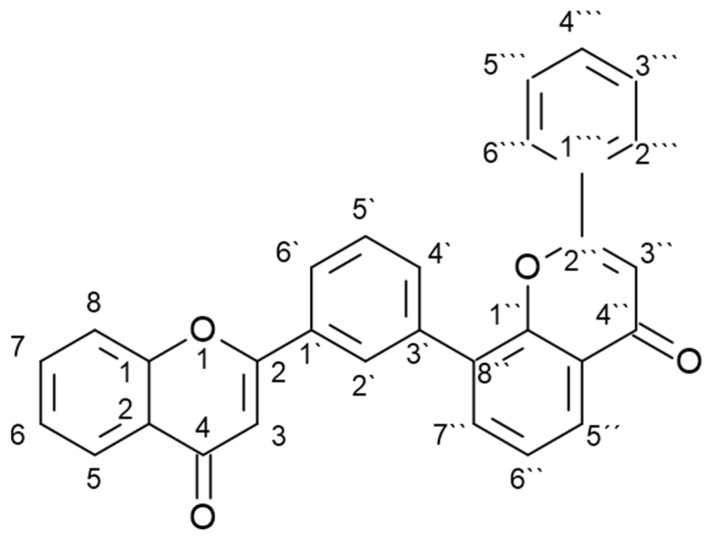
Basic structure of 3′, 8″- biflavones.

A plant in which various biflavonoids have been detected is the ginkgo (*Ginkgo biloba* L.), also called maidenhair tree [3,10]. It is a deciduous gymnosperm tree (family Ginkgoaceae) native to China and has been planted in Chinese and Japanese temple gardens since ancient times, but is now found as an ornamental tree in many parts of the world [11]. Its use in traditional Chinese medicine is well known, but today its extracts are widely used worldwide for the treatment of cognitive complaints [12]. Various specialized metabolites have been detected in ginkgo plant samples and extracts, including 110 different flavonoids, 13 of which are biflavonoids [3,10]. Although the presence of other biflavonoids has also been reported, the most commonly reported biflavonoids include amentoflavone, bilobetin, ginkgetin, isoginkgetin, and sciadopitysin [3]. These are all 3′, 8″- biflavones whose basic structure is shown in Figure 1.

The flavonoids in ginkgo are being researched by many scientists. However, most studies focus on monomeric flavonoids and biflavonoids are often neglected [13,14,15], e.g., in the review article by Liu et al. [16], which focuses on the advances in chemical analysis and quality control of flavonoids in ginkgo, the presence of biflavonoids is hardly mentioned. To fill this gap in our knowledge of ginkgo flavonoids, the aim of the present study was to establish a tissue-specific profile of biflavonoids in ginkgo. To this end, we developed the HPLC-DAD method that allowed us to simultaneously determine five biflavonoids, amentoflavone, bilobetin, ginkgetin, isoginkgetin, and sciadopitysin. We determined their content in ten different ginkgo plant parts and discussed their presence in relation to possible physiological functions.

## 2. Results

### 2.1. Chemical Differences and Method for Biflavonoids Analysis

**Figure 2:**
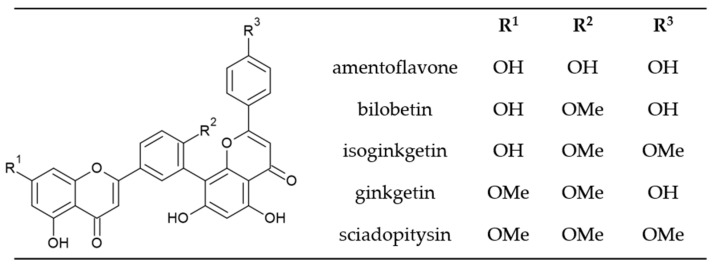
Chemical structure of main biflavonoids in ginkgo.

All five biflavonoids analyzed (Figure 2) belong to the amentoflavone type, in which two monomeric units are joined at positions 3′, 8″ (Figure 1). Amentoflavone consists of two apigenin monomers and other biflavonoids in ginkgo are considered derivatives of amentoflavone.

**Figure 3:**
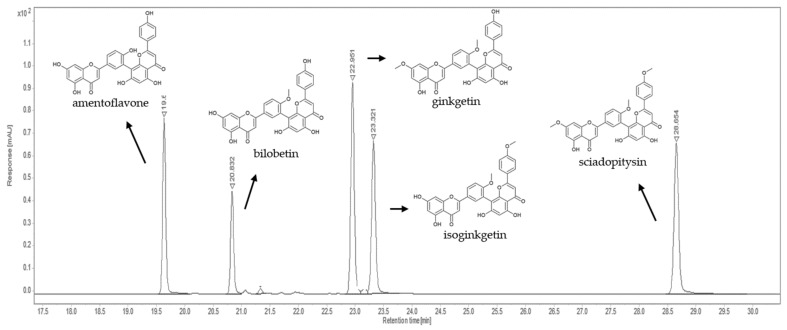
Representative chromatogram of five biflavones recorded at 330 nm.

Bilobetin has a methoxy group at the C-4′ position. Isoginkgetin and ginkgetin have two methoxy groups–isoginkgetin at the C-4′ and C-4‴ positions and ginkgetin at C-7 and C-4′. Sciadopitysin has three methoxy groups at C atom positions C-7, C-4′ and C-4‴. The presence of methoxy groups affects the chemical properties of the analyzed compounds and their polarity, which allows us to separate the compounds using reverse phase C 18 HPLC silica columns (Figure 3).

Under the proposed instrumental conditions, the biflavonoids eluted at 19.6 min (amentoflavone), at 20.8 min (bilobetin), at 23.0 min (ginkgetin), at 23.3 min (isoginkgetin), and at 28.7 min (sciadopitysin), as shown in Figure 3 and Table 1. Identification of each compound was performed by comparing the retention times and UV spectra of the analytes with those of the standard solution. According to the criteria of Commission Decision (EC) No. 657/2002 on the performance of analytical methods [17], the relative retention time of the determined compound in the sample solution had to be equal to that in the standard solution with a tolerance of ±2.5%. Quantification was performed using an external standard calibration and the amount of biflavonoids in the plant samples was expressed in µg/g dry weight (dw).

The limit of detection (LOD) and limit of quantification (LOQ) were estimated using the signal-to-noise (S/N) approach, calculated for the sample extract at the targeted level of 0.3 µg/mL for LOD and 1.0 µg/mL for LOQ, for each compound. The obtained S/N values were regarded as acceptable if ≥3 and ≥10, respectively. The achieved R^2^ of all calibration curves was greater than 0.99, and the targeted LOD and LOQ values for all five compounds (0.3 µg/mL and 1.0 µg/mL, respectively) were evaluated as acceptable, as satisfying the requirements of sufficient S/N ratios (Table 2).

### 2.2. Tissue-Specific Biflavonoids Profiling

**Figure 4:**
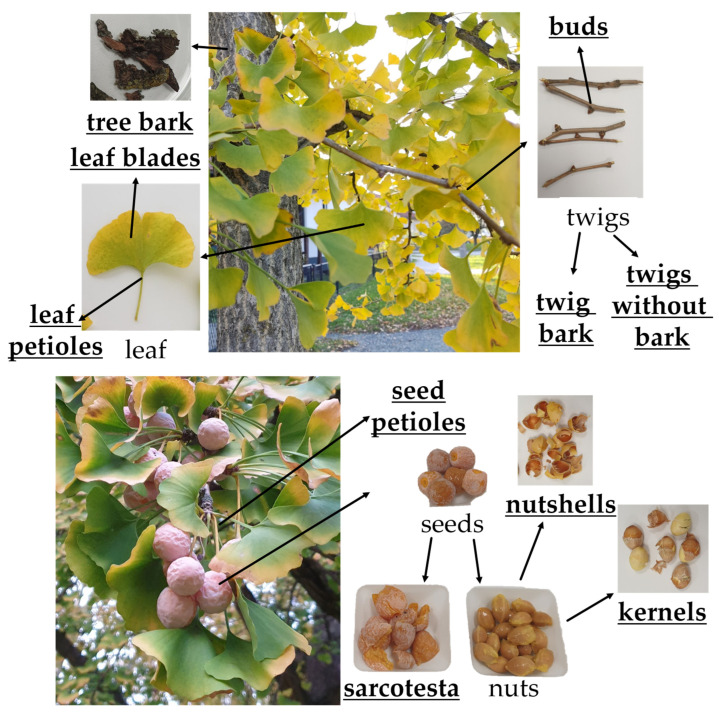
Ginkgo plant parts. Analyzed parts are bold and underlined.

To establish a tissue-specific biflavone profile, we separated the different ginkgo plant parts as shown in Figure 4. Various names have been used for the ginkgo seeds, which are sometimes called fruits, although the ginkgo belongs to the gymnosperms and produces seeds. The fleshy part is the seed coat or sarcotesta.

We can divide the analyzed plant parts into parts that are in direct contact with the environment, such as leaves (petiole and leaf blade), bark, twig bark, bud, seed petiole and sarcotesta, and parts that are not in direct contact with the environment, such as twig without bark, nutshell, and kernel. We did not detect biflavonoids in the parts that are not in direct contact with the environment.

**Figure 5:**
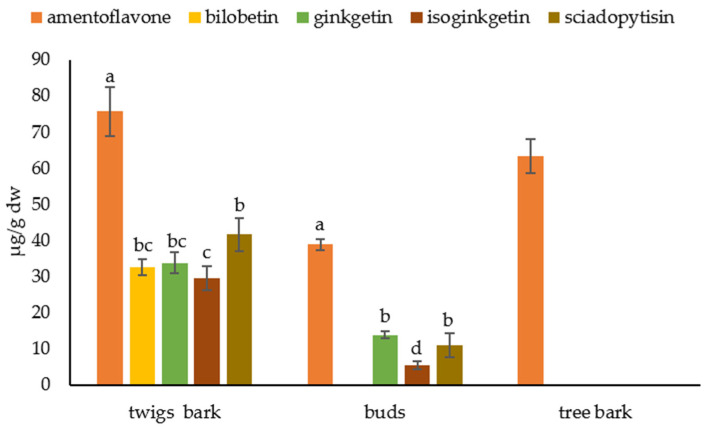
Biflavonoid profile in tree and twig bark and buds. Biflavonoid levels labeled with different letters differ significantly at *p* < 0.05 within the plant part.

Figure 5 shows the biflavonoid profile of tree and twig barks and buds. In tree bark, we find only amentoflavone at a concentration of 63.30 ± 4.60 µg/g dw. Amentoflavone was also the most abundant biflavonoid in twig bark and buds with a concentration of 75.70 ± 6.80 µg/g dw and 38.82 ± 1.55 µg/g dw, respectively. Other biflavonoids were also present in the twig bark: bilobetin in concentration 32.53 ± 2.30 µg/g dw, ginkgetin 33.79 ± 2.80 µg/g dw, isoginkgetin 29.49 ± 3.23 µg/g dw and sciadopitysin 41.57 ± 4.64 µg/g dw. In buds, we could not detect bilobetin, and ginketin, isoginkgetin, and sciadopitysin were found at concentrations of 13.79 ± 1.00 µg/g dw, 5.47 ± 1.03 µg/g dw, and 10.96 ± 3.43 µg/g dw, respectively.

**Figure 6:**
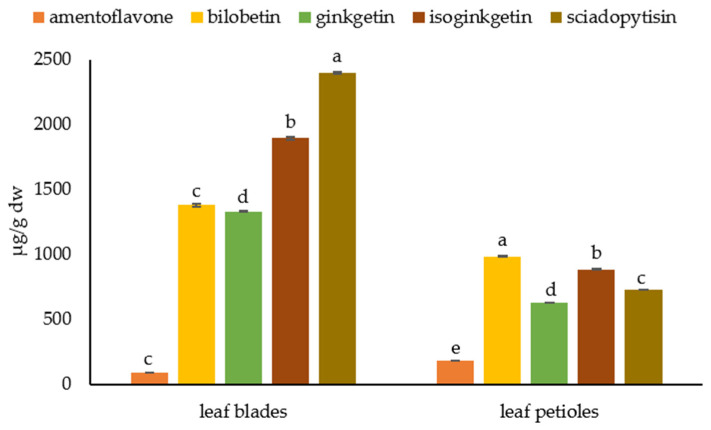
Biflavonoid profile in leaf parts- leaf blade and petiole. Biflavonoid levels labeled with different letters differ significantly at *p* < 0.05 within the plant part.

We found the highest content of biflavonoids in the leaf petioles and blades (Figure 6). In contrast to the barks, amentoflavones were the least abundant biflavonoids in the leaves (183.57 ± 1.18 µg/g dw in petioles and 86.00 ± 0.74 µg/g dw in leaf blades). In petioles, the most abundant biflavonoid was bilobetin (984.48 ± 6.42 µg/g dw), followed by isoginkgetin (883.83 ± 5.41 µg/g dw), sciadopitysin (727.14 ± 2.98 µg/g dw), and ginkgetin (627.22 ± 3.21 µg/g dw). In leaf blades, the most abundant biflavonoid was sciadopitysin (2398.59 ± 6.11 µg/g dw), followed by isoginkgetin (1896.02 ± 11.92 µg/g dw) and bilobetin and ginkgetin, whose contents were similar and were 1378.34 ± 11.22 µg/g dw and 1331.17 ± 5.85 µg/g dw, respectively.

**Figure 7:**
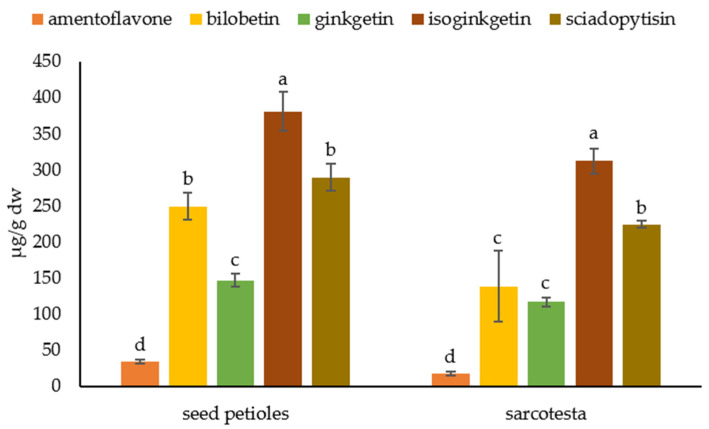
Biflavonoid profile of the seed parts. Biflavonoid levels labeled with different letters differ significantly at *p* < 0.05 within the plant part.

We analyzed seed petioles, sarcotesta, nutshells, and kernels separately and detected biflavonoids only in seed petioles and sarcotesta (Figure 7). In seed petioles, the most abundant biflavonoid was isoginkgetin (380.41 ± 26.98 µg/g dw), while the least abundant biflavonoid was amentoflavone (34.01 ± 2.51 µg/g dw). Other biflavonoids were present in the following concentrations: ginkgetin 146.62 ± 9.37 µg/g dw, bilobetin 249.29 ± 19.23 µg/g dw and sciadopitysin 289.32 ± 19.19 µg/g dw. In a sarcotesta, the biflavonoids were present in similar proportions. The largest abundant was isoginkgetin (311.67 ± 16.92 µg/g), followed by sciadopitysin (224.32 ± 4.44 µg/g dw), bilobetin (138.34 ± 49.19 µg/g dw), and ginkgetin (116.53 ± 6.58 µg/g dw). The least abundant biflavonoid in sarcotesta was amentoflavone with a concentration of 17.52 ± 2.77 µg/g dw.

**Figure 8:**
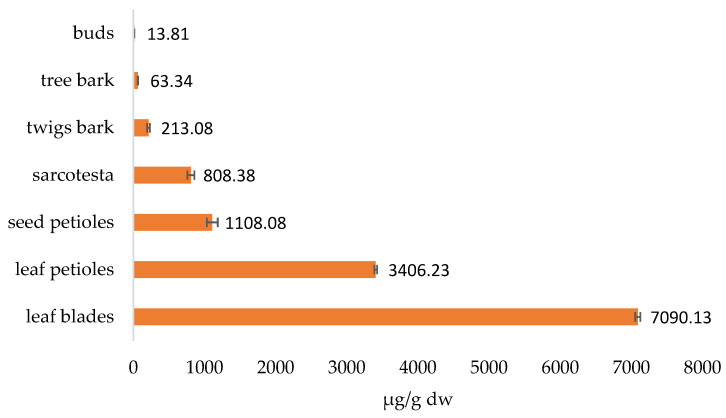
Total biflavonoid content in analyzed ginkgo parts.

The total content of biflavonoids in different parts of the ginkgo plant is shown in Figure 8. As you can see, the biflavonoid content is highest in the leaves, followed by the seeds, while the other parts have much lower biflavonoid content.

## 3. Discussion

In the past, separation and especially identification of biflavonoids was a difficult task due to lack of modern analytical equipment and commercially available standards. Many of the early reported methyl biflavones had to be corrected for structure, as structural elucidation in the 1960s and 1970s depended on co-chromatography with isolated authentic compounds, which in turn could be misidentified [18]. Biflavonoids have a strong signal at 330 nm, making the DAD detector in combination with standards a compelling and rapid method for detecting biflavonoids in ginkgo. One of the earliest HPLC-DAD methods for separating biflavonoids from ginkgo leaves was developed by Briancon-Scheid et al. [19], the HPLC-DAD method for the simultaneous determination of four (bilobetin, ginkgetin, isoginkgetin, sciadopitysin) biflavonoids. Kaur et al. [20] developed a method for the simultaneous determination of three biflavonoids (bilobetin, sciadopitysin, and ginkgetin) from ginkgo leaves, and recently Lei et al. [21] focused on four biflavonoids (bilobetin, ginkgetin, isoginkgetin, sciadopitysin), as did Wang et al. [22]. Pandey et al. [23] reported only two biflavonoids (amentoflavone and sciadopitysin) in a crude extract of ginkgo leaves. Beck and Stengel [7] like us, reported five biflavonoids in ginkgo leaves but were unable to separate ginkgetin from isoginkgetin. To determine the content of biflavonoids in different plant parts (tissues) of ginkgo, we have developed the HPLC-DAD method for the separation and simultaneous quantification of the five major biflavonoids in ginkgo [3], which allows us to profile different tissue types.

We analyzed ten plant parts and could not detect any of the analyzed biflavonoids in three parts (twigs without bark, nutshells, and seeds). Chen et al. [24] also failed to detect biflavonoids in seeds or embryoids, as they called this the seed part. We detected biflavonoids in all parts studied that are in contact with the environment, which may indicate a biological role for biflavonoids in the context of plant-environment interactions. This is consistent with literature data that biflavonoids are localized in the outer part of the leaves of *G. biloba* [7] and above the ground rhizome of *Psilotum nudum* [8] according to MALDI imaging data.

We found the highest total biflavonoid content and the presence of all five biflavonoids in the leaf blades, followed by the petioles. In the leaf blades, the most abundant biflavonoid was sciadopitysin, which is consistent with the report of Wang et al. [22] who also described sciadopitysin as the most abundant biflavonoid in yellow ginkgo leaves. In leaf parts, we detected a much lower amount of amentoflavone compared with other biflavonoids, suggesting that more methylated biflavonoids are present in leaves. A similar high amount of sciadopitysin compared to amentoflavone in leaves was reported by Pandey et al. [23] in ginkgo trees of different ages. The low content of amentoflavone is probably the reason why this biflavonoid was generally not detected in leaves in other studies [19,21,22]. Amentoflavone was also the least abundant biflavonoid in the seed parts where biflavonoids were detected in seed petioles and sarcotesta, according to our data. Chen et al. [24] reported only isoginkgetin in ginkgo sarcotesta at concentrations ranging from 179.78 to 424.42 µg/g dry weight, which is comparable to our results where isoginkgetin was the most abundant biflavonoid (380.41 ± 26.98 µg/g dw). Shen et al. [25] reported bilobetin, isoginkgetin, and ginkgetin in sarcotesta and developed a method for large-scale targeted isolation of these biflavonoids from the exocarp (sarcotesta), which is an industrial waste.

In contrast to the leaves and seeds, in which methylated biflavonoids dominate, the most abundant biflavonoid in the bark and buds of trees and twigs was amentoflavone. In the bark, we could detect only amentoflavone and at the same time, other biflavonoids were below the detection limit. In the bark of the twig, we also detected other biflavonoids, but the amentoflavone content was twice as high as in other parts. Pandey et al. [23] reported the presence of amentoflavone and sciadopitysin in ginkgo stems, but the authors did not specify which type of stems they analyzed. The presence of amentoflavone has also been reported in the bark of other plants such as *Calophyllum pinetorum* [26], *Ochna schweinfurthiana* [27] and *Anacolosa poilanei* [28]. If we consider the strong antimicrobial [29,30] and antiparasitic [31,32,33] activity of amentoflavone, this could indicate its role in protection against biotic stress, but further studies should test this hypothesis.

## 4. Materials and Methods

### 4.1. Materials and Plant Samples

Ultrapure water was prepared using the Purelab flex system (ELGA LabWater, Wycombe, UK) and all solvents used were of HPLC grade. Certified analytical standards of biflavonoids, including amentoflavone (CAS 1617-53-4), bilobetin (CAS 521-32-4), ginkgetin (CAS 481-46-9), isoginkgetin (CAS 548-19-6), and sciadopitysin (CAS 521-34-6), were purchased from Phytolab (Vestenbergsgreuth, Germany). Plants of *Ginkgo biloba* L. were collected in Koprivnica, Croatia, in late October 2022. Leaves, seeds and bark samples were collected and then separated to obtain different tissues (Figure 4). Fresh material was quickly frozen and freeze-dried using a laboratory freeze dryer (LIO-5PLT, KAMBIČ, Ljubljana, Slovenia). The dry plant material was pulverized using a bead mill (Bead Ruptor 12, Omni International, USA), sealed under vacuum and stored at room temperature for further experiments.

### 4.2. Preparation of Standard Solutions

Standard stock solutions of five biflavonoids (amentoflavone, bilobetin, sciadopitysin, ginkgetin and isoginkgetin) were prepared individually in DMSO in a concentration of 1000 µg/mL. An appropriate amount of each standard stock solution was diluted with 80% methanol to obtain seven working standard solutions (concentrations 1.0, 2.0, 5.0, 10, 20, 50 and 100 µg/mL) for constructing the relevant calibration curves. The standard stock solution, along with the working solutions, was kept in dark at −20 °C until use.

### 4.3. Biflavonoids Extraction

Powdered samples of various *G. biloba* L. plant parts were accurately weighed (30 mg) and sonicated for 10 min at room temperature in the presence of extraction solvent (1 mL of 80% methanol), followed by a 45 min rotation in a mechanical shaker (Bio RS-24, Biosan, Latvia) and 5 min centrifugation (LMC-4200R, Biosan, Latvia) at 4000× *g*. Prior to the instrumental analysis, the obtained sample extracts were filtered through a 45 µm pore size polytetrafluoroethylene syringe filter. Each plant sample type was prepared in triplicates after which the average result value was expressed for each biflavonoid compound.

### 4.4. HPLC-DAD Analysis

The instrumental analysis was performed using an Agilent 1260 Infinity II HPLC system (Agilent, Santa Clara, CA, USA), equipped with a quaternary pump system, autosampler, column department and diode array detector (DAD). The acquisition and data processing was conducted using Agilent OpenLAB CDS software (v. 2.6, Agilent, Santa Clara, CA, USA). Chromatographic separation was attained using Zorbax 300Extend-C18 column, 150 × 4.6, 3.5 µm (Agilent, Santa Clara, CA, USA) maintained at 30 °C, and 0.1% formic acid in water (mobile phase A) and acetonitrile (mobile phase B) at a constant flow rate of 1.0 mL/min during 42 min of the analysis. The used multistep linear solvent gradient for the analyte’s elution was as follows: 0 min 98% A, 5 min 90% A, 15 min 70% A, 20 min 50% A, 25 min 50% A, 30 min 20% A, 32 min 2% A, 40 min 98% A. The injection volume was 10 µL for both standards and samples. UV/Vis spectra were recorded in the range of 190–400 nm, and the chromatograms were acquired at 330 nm.

### 4.5. Statistical Analysis

For analysis, we collected samples from six different plants, three of which were female from which we took seeds. We all collected material pulled together and for analysis, we performed three separate extractions and analyzed them on HPLC-DAD. Statistical analyses were performed using the free software PAST [34]. One-way ANOVA and post hoc multiple means comparison (Tukey’s HSD test) were performed and differences between measurements were considered significant at *p* < 0.05.

## 5. Conclusions

In the present study, we developed a method for the simultaneous determination of five biflavonoids (amentoflavone, bilobetin, ginkgetin, isoginkgetin, and sciadopitysin) and performed tissue-specific profiling in ten different parts of ginkgo plants. We did not detect biflavonoids in three plant parts (twigs without bark, nutshells, and kernels) that were not in direct contact with the environment. In the other plant parts studied, biflavonoids were most abundant in leaves, followed by seeds. In bark samples and buds, the predominant biflavonoid was amentoflavone, which, in contrast, was least abundant in leaves and seeds, where methylated biflavonoids dominated. In petioles and leaf blades, the most abundant biflavonoid was sciadopitysin, and in petioles and the sarcotesta of seeds, isoginkgetin. The accumulation of biflavonoids in the parts in direct contact with the environment probably indicates their role in plant-environment interaction. However, the different accumulation of each biflavonoid might be related to their chemical structure and different biological functions. Further studies should focus on elucidating the role of biflavonoids in plants, with emphasis on the structural functional differences.

## Figures and Tables

**Table 1:** Retention times and UV spectrum data for analyzed biflavonoids.

Compound	Retention Time (min)	Characteristic UV Spectrum	Maximum UV Apsorption (nm)
Amentoflavone	19.634	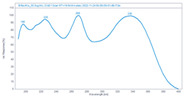	196, 226, 268, 336
Bilobetin	20.832	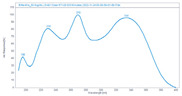	196, 230, 270, 334
Ginkgetin	22.951	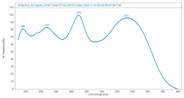	196, 228, 270, 332
Isoginkgetin	23.321	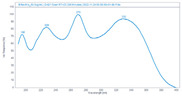	196, 228, 270, 330
Sciadopitysin	28.654	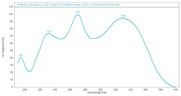	194, 232, 270, 330

**Table 2:** Curve equation, R^2^, LOD and LOQ for analyzed biflavonoids.

Analyte	Wavelength nm	Curve Equation	R^2^	LOD µg/mL	LOQ µg/mL
Amentoflavone	330	y = 36.7275x − 51.0679	0.99504	0.30	1.0
Bilobetin	330	y = 23.2259x − 33.033	0.99758	0.30	1.0
Ginkgetin	330	y = 54.6868x − 74.7767	0.99765	0.30	1.0
Isoginkgetin	330	y = 44.1283x − 51.4203	0.99775	0.30	1.0
Sciadopitysin	330	y = 49.3575x − 59.0710	0.99806	0.30	1.0

## Data Availability

Not applicable.
